# Neuromyelitis Optica Spectrum Disorder Initially Presenting With Meningitis-Like Symptoms: A Case Report

**DOI:** 10.7759/cureus.101638

**Published:** 2026-01-15

**Authors:** Yuki Kubo, Takuya Nishimura, Chinatsu Sakuragi, Junki Yoshimura, Seiji Okubo

**Affiliations:** 1 Neurology, NTT Medical Center Tokyo, Tokyo, JPN

**Keywords:** anti-aquaporin-4 antibody (anti-aqp4 antibody), aseptic meningitis, case report, cerebrospinal fluid, hypoglycorrhachia, meningitis, neuromyelitis optica spectrum disorder (nmosd), optic neuritis, relapsing nmosd, visual impairment

## Abstract

We report a case of neuromyelitis optica spectrum disorder (NMOSD) relapse in a 45-year-old woman with a known 11-year history of NMOSD, who initially presented with meningitis-like symptoms, including fever, headache, and neck stiffness. Physical examination revealed neck stiffness and positive jolt accentuation, without any other neurological abnormalities. Cerebrospinal fluid (CSF) analysis revealed mononuclear-predominant pleocytosis and hypoglycorrhachia, raising concerns of meningitis. On hospital day 2, brain magnetic resonance imaging (MRI) revealed new hyperintense lesions in the right lateral midbrain and bilateral subcortical regions. That night, the patient developed acute right-sided visual impairment. The patient was diagnosed with NMOSD relapse and was treated with intravenous methylprednisolone pulse therapy, achieving gradual visual improvement.

This case highlights that NMOSD can initially present with meningitis-like symptoms, and CSF profiles may resemble those of infectious meningitis. Clinicians should therefore maintain diagnostic vigilance for NMOSD in patients presenting with meningitis-like symptoms, particularly in those with a prior diagnosis of NMOSD or nonadherence to treatment.

## Introduction

Neuromyelitis optica spectrum disorder (NMOSD) is a rare, severe autoimmune inflammatory disorder of the central nervous system, with a worldwide prevalence of approximately 0.3 to 4.4 per 100,000 individuals [1]. The disorder predominantly affects women, with a female-to-male ratio of approximately 9:1 in anti-aquaporin-4 (AQP4) antibody-seropositive cases [2]. Approximately 70%-80% of patients with NMOSD are seropositive for AQP4 antibodies, which target water channels on astrocytes and play a central role in disease pathogenesis [3].

NMOSD typically manifests with optic neuritis, longitudinally extensive transverse myelitis, area postrema syndrome, and other brainstem or diencephalic syndromes [3]. Cerebrospinal fluid (CSF) analysis during acute attacks commonly reveals pleocytosis, which can be neutrophil-predominant in the early phase, along with elevated protein levels [4-7]. Although hypoglycorrhachia is uncommon in NMOSD, it has been reported in some cases and may mimic bacterial meningitis, potentially leading to diagnostic delays.

Meningitis-like presentations, characterized by fever, headache, and meningeal signs, are rare but increasingly recognized manifestations of NMOSD. In this report, we presented a case of NMOSD relapse initially presenting as meningitis, which subsequently progressed to rapid visual loss within 24 hours of admission and reviewed previously reported cases to highlight the clinical features of this atypical presentation.

## Case presentation

The patient was a 45-year-old woman who presented with headache as the chief complaint. She had a significant medical history: at 19 years of age, she experienced a transient visual field disturbance. At the age of 32, she developed transient sensory symptoms following aseptic meningitis and was diagnosed with multiple sclerosis. At 34 years of age, she experienced a relapse of myelitis during pregnancy, tested positive for AQP4 antibodies, and was rediagnosed with NMOSD. She remained relapse-free on oral prednisolone 5 mg/day but had recently become treatment non-adherent.

On illness day 1, she developed rhinorrhea, sputum production, and cough. Fever (38.2°C) appeared on day 2, without improvement after acetaminophen. On day 7, she received symptomatic treatment at a local clinic. In the early morning of day 8, she developed a severe headache and consequently presented to our hospital.

Physical examination at admission revealed the following vital signs: height, 160 cm; weight, 46 kg; blood pressure, 93/64 mmHg; pulse, 49 bpm; and temperature, 36.5°C. General physical examination results were unremarkable.

Neurological examination revealed that the patient was alert and well-oriented; however, she exhibited neck stiffness and positive jolt accentuation. Cranial nerve, motor, sensory, cerebellar, and reflex examinations revealed no abnormalities. She had constipation but had normal urination.

Laboratory and imaging findings were as follows (Table 1): blood tests revealed leukocytosis (10,300/μL) and elevated C-reactive protein (CRP) (3.85 mg/dL). CSF analysis revealed a white blood cell (WBC) count of 126/mm³ (51% mononuclear), a protein level of 241 mg/dL, and a glucose level of 36 mg/dL (serum glucose, 97 mg/dL). Head computed tomography (CT) was normal; therefore, a provisional diagnosis of aseptic meningitis was established. The CSF herpes simplex virus-polymerase chain reaction (HSV PCR) results were negative.

**Table 1:** Laboratory and cerebrospinal fluid findings on admission WBC, white blood cell; CRP, C-reactive protein; CSF, cerebrospinal fluid; HSV PCR, herpes simplex virus polymerase chain reaction

Laboratory parameter	Result	Reference range
Peripheral blood		
WBC (/μL)	10,300	3,300-8,600
CRP (mg/dL)	3.85	<0.14
Serum glucose (mg/dL)	97	73-109
Cerebrospinal fluid		
WBC (/mm³)	126	0-5
Mononuclear cells (%)	51	-
Protein (mg/dL)	241	15-45
Glucose (mg/dL)	36	50-75
CSF/serum glucose ratio	0.37	>0.6
HSV PCR	Negative	Negative

**Figure 1:**
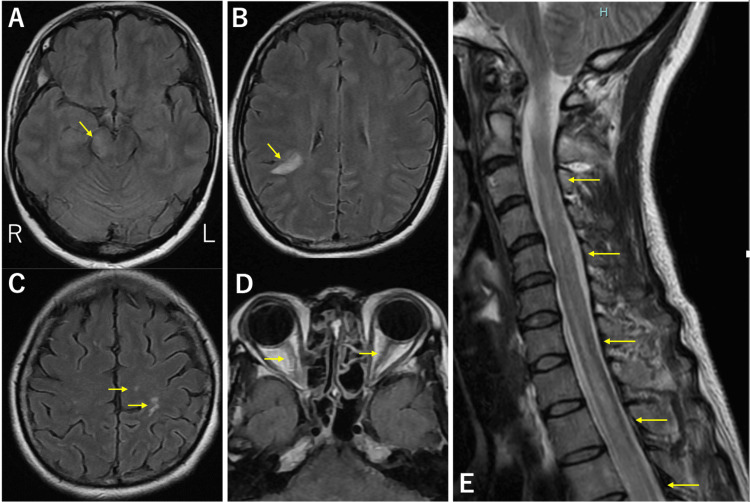
MRI of the brain and spinal cord FLAIR images show high signal intensity in the right lateral midbrain (arrow, A: axial, 1.5T) and bilateral subcortical areas (arrows, B, C: axial, 1.5T). No obvious bilateral abnormalities were observed in the optic nerves (arrows, D: axial, 1.5T). Cervical spine T2-weighted images show hyperintensity along the central canal from the C3 to T2 vertebral levels (arrows, E: sagittal, 1.5T). FLAIR, fluid-attenuated inversion recovery; MRI, magnetic resonance imaging

Acyclovir (1500 mg/day) was initiated empirically on hospital day 1 to rule out HSV encephalitis, given the acute presentation with fever and neurological symptoms. Empirical antibiotics were withheld because bacterial meningitis was considered unlikely, based on the mononuclear-predominant pleocytosis and relatively stable general condition. On day 2, brain magnetic resonance imaging (MRI) revealed new fluid-attenuated inversion recovery (FLAIR) hyperintense lesions in the right lateral midbrain and bilateral subcortical regions, without optic nerve abnormalities (Figure 1).

On the morning of day 2 of hospitalization, the patient noticed decreased visual acuity in the right eye. Ophthalmology consultation on day 3 revealed visual acuity of 0.02 (uncorrectable) OD and 0.1 (corrected to 1.2) OS. Given the risk of irreversible visual loss, high-dose intravenous methylprednisolone (1 g/day for five days) was initiated promptly without waiting for HSV PCR results, which later returned negative on hospital day 7.

**Figure 2:**
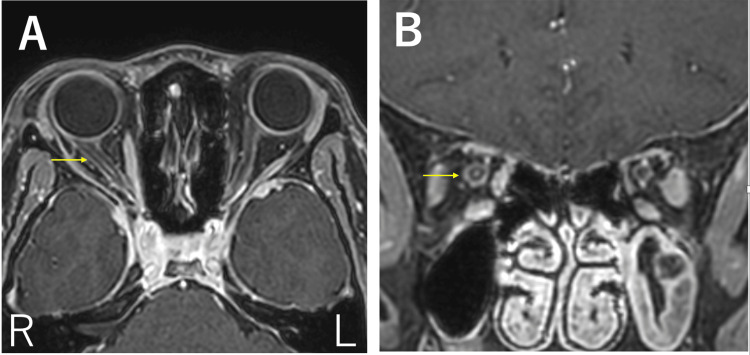
Contrast-enhanced brain MRI on hospital day 7 T1-weighted images show right optic nerve enlargement with internal contrast enhancement (arrows, A: axial; B: coronal, 1.5T). MRI, magnetic resonance imaging

Cervical spine MRI revealed new longitudinal T2 hyperintensities from C3 to T2 (Figure 1). Contrast-enhanced MRI on day 7 demonstrated enhancement of new lesions and the right optic nerve (Figure 2).

By day 9, the cervical MRI abnormalities had decreased, and a repeat CSF examination showed an improvement in cell count and protein levels. Despite treatment, right eye visual acuity deteriorated to hand motion by hospital day 7. Owing to persistent visual impairment, a second steroid pulse was administered starting on day 10. Prednisolone was then tapered from 40 mg/day. At discharge, visual acuity recovered to 0.02 OD and 1.2 OS (corrected). Although some recovery was observed, significant visual impairment persisted in the right eye.

Anti-AQP4 antibody testing returned positive results, confirming NMOSD relapse. She was discharged on hospital day 18 and restarted relapse-prevention therapy with inebilizumab 300 mg on illness day 86. Over one year of follow-up, the patient remained relapse-free, with gradual visual recovery, and returned to work.

## Discussion

In this report, we described a case of NMOSD relapse, initially presenting with meningitis-like symptoms and acute visual loss within 24 hours of admission.

At initial presentation, NMOSD relapse was not immediately suspected despite her known history, for several reasons: (1) her presenting symptoms - fever, headache, and meningeal signs without focal neurological deficits - were more suggestive of infectious meningitis than typical NMOSD relapse; (2) classic NMOSD manifestations, such as optic neuritis or transverse myelitis, were absent at admission; and (3) the CSF profile, with hypoglycorrhachia, initially raised strong concern for bacterial meningitis. NMOSD relapse became the leading differential only after the emergence of visual impairment on hospital day 3.

The differential diagnosis at admission included viral meningitis/encephalitis (particularly HSV), autoimmune or aseptic meningitis, NMOSD relapse with meningeal involvement, and bacterial meningitis. Although hypoglycorrhachia raised concern for bacterial etiology, viral meningitis was considered the most likely diagnosis, based on the mononuclear-predominant pleocytosis and relatively stable general condition. Therefore, acyclovir was initiated empirically, while antibiotics were withheld. The emergence of visual impairment and longitudinally extensive myelitis ultimately confirmed NMOSD relapse.

Typical CSF findings in NMOSD include polymorphonuclear-predominant pleocytosis, elevated protein levels, and normal or reduced glucose [4-7]. These findings closely mimicked bacterial meningitis [8]. Hypoglycorrhachia in NMOSD may result from impaired glucose transport across the blood-brain barrier, due to AQP4-targeted immune activity [9], and increased glucose consumption by inflammatory cells [10].

Interestingly, our case demonstrated mononuclear-predominant pleocytosis, which contrasts with the polymorphonuclear predominance reported in most previous NMOSD cases with meningitis-like presentation (Table 2). Polymorphonuclear pleocytosis typically suggests acute bacterial infection, while mononuclear predominance is more consistent with viral or autoimmune etiologies. The mononuclear pattern in our case, combined with negative bacterial cultures and HSV PCR, provided an early clue favoring a non-infectious process, though hypoglycorrhachia initially complicated the differential diagnosis. Oligoclonal band testing was not performed during this admission. To date, only 13 cases of NMOSD initially presenting with meningitis have been reported [8-18].

**Table 2:** Features of meningitis-like presentation in NMOSD NMOSD, neuromyelitis optica spectrum disorder; M, male; F, female; CSF, cerebrospinal fluid; WBC, white blood cell; Poly, polymorphonuclear cell; Mono, mononuclear cell; MRI, magnetic resonance imaging

Reference	M/F	Age	NMO phase	Fever headache meningism	Neurological symptoms on admission	CSF WBC (/μL)	CSF cell differential	CSF protein (mg/dL)	CSF glucose (mg/dL)	MRI findings	First MRI (admission day)
Saab et al. [15]	F	49	First onset	+	Quadriplegia	12565	Polynuclear	296	<10	Cerebrum, cervical, thoracic spine	1
Li et al. [13]	F	31	First onset	+	Paraplegia	1280	Polynuclear	3835	32	Cervical spine	4
Lepur et al. [8]	F	47	First onset	+	Whole body pain	540	Polynuclear	103	29	Cervical, thoracic spine	-
Lepur et al. [8]	M	60	First onset	+	Lower back abdominal pain	4864	Polynuclear	309	29	Cervical, thoracic spine	-
Wang et al. [17]	M	40	First onset	+	Disturbance of consciousness	606	Mononuclear	102	Normal	Cerebrum, diencephalon, brainstem	-
Wang et al. [17]	F	38	First onset	+	Disturbance of consciousness, nystagmus	625	Mononuclear	285	30	Cerebrum, diencephalon, cerebellum	-
Shi et al. [16]	F	28	First onset	+	Transverse myelitis	280	Polynuclear	3680	12	Cerebrum, spine	-
Shi et al. [16]	F	34	First onset	+	Transverse myelitis	1200	Polynuclear	2151	32	Spine	-
He et al. [12]	F	29	Recurrence	+	Quadriplegia, impaired right vision	1131	Polynuclear	1587	40	Spine	2
Bu et al. [11]	F	54	First onset	+	Disturbance of consciousness, convulsions	281	-	114	45	Nothing	3
Zhang et al. [18]	M	30	First onset	+	-	205	Polynuclear	67	34	Nothing	-
Fujikura et al. [10]	M	57	First onset	+	Complete paraplegia	1719	Polynuclear	420	20	Cervical spine	1
Odachi et al. [14]	F	20	First onset	+	-	69	Mononuclear	44	48	Diencephalon, brainstem, spine	4
Our case	F	45	Recurrence	+	-	126	Mononuclear	241	36	Cerebrum, diencephalon, spine	2

All patients exhibited fever, headache, and meningeal irritation, and 11 showed additional focal neurological signs. MRI abnormalities were identified within four days in nearly all reported cases. Only two cases presented with meningitis-like symptoms on admission.

Our case was diagnostically challenging because the CSF profile, with elevated protein and decreased glucose levels, closely resembled that of infectious meningitis. However, MRI lesions appeared on hospital day 2, and visual symptoms developed rapidly thereafter. Although steroid therapy was initiated promptly, visual recovery was delayed.

Given the high relapse rate of untreated NMOSD [19] and its potential severity [20], clinicians should consider NMOSD in patients with meningitis-like presentations, a known history of NMOSD, poor treatment adherence, or unexplained MRI lesions. Early steroid administration in such cases, even when treating suspected infections, may improve outcomes.

## Conclusions

NMOSD can initially mimic infectious meningitis, both clinically and in terms of the CSF profile, thus complicating early diagnosis. In patients with prior NMOSD, treatment noncompliance, or newly detected MRI lesions, clinicians should maintain a high index of suspicion for NMOSD relapse during the initial evaluation of meningitis-like presentations.
